# Comparison of psychometric properties between usual-week and past-week self-reported physical activity questionnaires: a systematic review

**DOI:** 10.1186/s12966-017-0470-6

**Published:** 2017-01-31

**Authors:** Kenji Doma, Renée Speyer, Anthony S. Leicht, Reinie Cordier

**Affiliations:** 1 College of Healthcare Sciences, James Cook University, Townsville, QLD Australia; 2 Department of Otorhinolaryngology and Head and Neck Surgery, Leiden University Medical Center, Leiden, The Netherlands; 3 School of Occupational Therapy and Social Work, Curtin University, Perth, WA Australia

**Keywords:** Physical activity questionnaires, Recall methods, Psychometrics, Validity, Reliability

## Abstract

The aim was to critically appraise the methodological quality of studies and determine the psychometric qualities of Past-week and Usual-week Physical Activity Questionnaires (PAQs). Data sources were obtained from Pubmed and Embase. The eligibility criteria for selecting studies included: 1) at least one psychometric property of PAQs was examined in adults; 2) the PAQs either had a recall period of usual 7-days (Usual-week PAQs) within the past 12 months or during the past 7-days (Past-week PAQs); and 3) PAQs were self-administered. Study quality was evaluated using the COSMIN taxonomy and the overall psychometric qualities evaluated using pre-established psychometric criteria. Overall, 45 studies were reviewed to assess the psychometric properties of 21 PAQs with the methodological quality of most studies showing good to excellent ratings. When the relationship between PAQs and other instruments (i.e., convergent validity) were compared between recall methods, Past-week PAQs appeared to have stronger correlations than Usual-week PAQs. For the overall psychometric quality, the Incidental and Planned Exercise Questionnaire for the Usual-week (IPEQ-WA) and for the Past-week (IPEQ-W) had the greatest number of positive ratings. For all included PAQs, very few psychometric properties were assessed with poor ratings for the majority of the overall qualities of psychometric properties indicating the limitation of current PAQs. More research that covers a greater spectrum of psychometric properties is required to gain a better understanding of the qualities of current PAQs.

## Background

Increasing the level of physical activity (PA) is paramount for improving physical and psycho-social health across a wide range of populations [1]. In fact, physical inactivity is now considered to be one of the four leading risk factors for developing chronic disease and global mortality [2]. Subsequently, measuring the level of PA is important to ascertain at-risk populations and monitor interventions aimed at reducing chronic disease development. However, PA determination is only viable when implementing valid and reliable measures that: a) determine frequency, intensity and type of PA; b) identify individuals that meet health recommendations; and c) evaluate the effectiveness of various PA modalities on specific outcome measures [3].

Several objective measures of PA have been developed including accelerometers, pedometers and heart rate monitors [4]. Whilst these methods are considered valid and reliable for determining PA level [4], they are often too costly and/or cumbersome to use. Furthermore, the validity of accelerometer-based estimates of PA has also been called into question [5]. Prior to these objective measuring devices, subjective measures such as PA questionnaires (PAQs) were used to determine PA level and still remain the preferred method as they can be self-administered and convenient and cost-effective, particularly in large-scale clinical trials [6]. However, misreporting of PA is common with PAQs, particularly due to difficulties recalling the intensity and type of PA performed previously [7]. Subsequently, greater attention is needed to determine the quality of psychometric properties of a range of PAQs.

Currently, there are two main recall methods that determine previous PA level. The first method identifies recent PA level over the past 7 days (i.e., Past-week PAQs) [8]. The second method assesses average week PA level within the past 1–12 months (i.e., Usual-week PAQs) [9]. Both types of PAQs have several advantages and disadvantages. For example, Usual-week PAQs can provide habitual PA patterns minimising the inherent weekly variation in PA [10]. However, respondents may experience difficulty in recalling their PA patterns over a longer period of time, particularly at light-moderate intensities [11]. Conversely, Past-week PAQs result in more accurate recall of recent PA patterns and therefore may better represent objective measures [12]. However, Past-week PAQs do not account for week-to-week variability in PA level and thus may misclassify individuals as physically active/inactive. Therefore, Past-week and Usual-week PAQs provide distinct characteristics of PA which researchers need to consider when selecting PAQs for their intervention. Delbaere et al. [13] compared different recall versions (i.e., Past-Week [W] vs. Average Weekly PA over the past three months [WA]) of the Incidental and Planned Exercise Questionnaire (IPEQ) in older people noting that IPEQ-WA had better psychometric properties overall, with better internal consistency and higher test-retest reliability than the IPEQ-W. However, examination of convergent validity against objective measures (e.g., accelerometers, pedometers) was not conducted for each recall method of IPEQ, despite using objective measures considered as the best approach for establishing PAQ validity [14]. Furthermore, whilst [13] measured test-retest reliability, convergent validity, structural validity and internal consistency, they did not compare measurement error between IPEQ-W and IPEQ-WA and content validity was not addressed. In order to identify the delimitations of PAQs due to different recall methods, and to assist practitioners and researchers with the best selection of robust PAQs, all psychometric properties of PAQs should be evaluated.

The Consensus-based Standards for the selection of health Measurement Instrument (COSMIN) group developed a critical appraisal tool to evaluate the methodological quality of studies that examined the psychometric properties of health measurement instruments [15]. This appraisal tool, known as the COSMIN checklist, allows for determination of the quality of study design and statistical analyses on validity, reliability and responsiveness of questionnaires [15]. Silsbury et al. [16] recently examined the methodological quality of studies examining the psychometric properties of ten selected self-reported PAQs using the COSMIN checklist. The authors reported fair-to-good test-retest reliability of PAQs and variable convergent validity against other objective measures. Whilst these findings provide insight on the usability of the 10 selected PAQs, the authors did not provide a clear description of the inclusion/exclusion criteria used for selecting PAQs nor give consideration for PAQs recall methods which introduces bias. Furthermore, appropriate search strategies for literature database using ‘subject headings’ and ‘free texts’ were not reported, limiting the replicability of the searches. Moreover, [16] did not interpret the psychometric quality of PAQs based on an established quality criterion. Terwee et al. [17] developed a quality criterion to interpret results from studies assessing the psychometric properties of questionnaires based on previously existing guidelines and consensus amongst experts. Furthermore, [18] suggested synthesising and combining results from COSMIN rating of study quality and [17] rating of psychometric quality to report the overall quality of psychometric properties of each questionnaire.

Indeed, previous studies have used similar quality criteria to review the psychometric quality of self-reported PAQs [19–21]. However, these review papers appeared to have been derived by the same literature search and were separated according to PAQs for youth [20], adults [19] and the elderly [21]. Combining results of studies that have examined the psychometric qualities of PAQs amongst different population groups may provide a more holistic understanding of the usability of existing PAQs. Furthermore, the computerised search for these systematic reviews [19–21] was conducted in May 2009 and thus warrants an update considering the constant growing body of literature in psychometrics. Importantly, none of the systematic reviews published to date have systematically compared the quality of psychometric properties between PAQs with different recall methods (e.g., usual-week versus past-week PAQs) using previously established quality criteria.

Therefore, the aims of this systematic review were to critically appraise the methodological quality of studies that have examined the psychometric properties of past-week and usual-week PAQs in adult and elderly populations using the COSMIN checklist to determine the overall psychometric quality for each PAQ, and to compare the quality of measurement properties between past-week and usual-week PAQs. Identification of recall differences would substantially assist practitioners and researchers with their selection and implementation of robust and high quality PAQs.

## Methods

The methodology and reporting of this systematic review was based on the PRISMA guidelines which enables transparent and complete reporting of systematic reviews [22].

### Inclusion/exclusion criteria

The following inclusion criteria for studies were adhered to: 1) studies that examined at least one measurement property of PAQs used in adults (i.e., ≥ 18 years of age); 2) studies that were written in English; 3) studies that examined PAQs with a recall period of 7-days PA within the past 12 months (i.e., Usual-week PAQs) or studies that examined PAQ during the past 7-days (i.e., Past-week PAQs); 4) studies that examined self-administered PAQs; and 5) studies where the PAQ identified the following PA characteristics: duration, intensity and/or type of PA performed. Studies were excluded if: 1) questionnaires were based on physical function measures; 2) PAQs were administered as an interview; and 3) results were published as a conference abstract, review or case report. Studies were excluded if questionnaires were translated into a language other than English.

### Search strategy

A systematic literature search was conducted to identify all relevant studies examining the measurement properties of PAQs in adults. Two electronic data bases (Medline and EMBASE) were used with searches conducted between July 1st 2016 and July 15th 2016, using both free-text words and subject headings (Table 1). All primary sources (i.e., journal articles) up to July 2016 were considered as part of the search.

**Table 1:** Search terms and databases

Initial search: Assessment retrieval	Database and Search Terms	Limitations
Subject Headings	**Embase**: (Questionnaire/OR Health status/OR “severity of illness index”/) AND (Physical capacity/OR “physical constitution and health”/OR “movement (physiology)”/OR “physical activity, capacity and performance”/OR Exercise/OR Performance/OR Motor performance/) AND (Validation study/OR validity/OR Psychometry/OR Reliability/OR Measurement accuracy/OR measurement error/OR measurement precision/OR measurement repeatability/)	Humans; English; Adult: 18 to 64 years OR Aged: 65+ years
	**PubMed**: (“Physical Conditioning, Human”[Mesh] OR “Physical Fitness”[Mesh] OR “Physical Therapy Modalities”[Mesh] OR “Physical Endurance”[Mesh] OR “Physical Exertion”[Mesh] OR “Exercise”[Mesh] OR “Motor Activity”[Mesh] OR “Exercise”[Mesh] OR “Exercise Movement Techniques”[Mesh] OR “Exercise Therapy”[Mesh] OR “Psychomotor Performance”[Mesh] OR “Motor Skills”[Mesh] OR “Motor Activity”[Mesh]) AND (“Questionnaires”[Mesh]) AND (“Psychometrics”[Mesh] OR “Reproducibility of Results”[Mesh] OR “Validation Studies as Topic”[Mesh] OR “Bias (Epidemiology)”[Mesh] OR “Observer Variation”[Mesh])	Humans; English; Adult: 19+ years
Free Text Words	**Embase** *:* (questionnaire*) AND (physic* OR movement* OR capacit* OR exercise* OR train* OR performance* OR motor) AND (psychometric* OR reliability OR validit* OR reproducibility OR bias)	Publication date from 2013 – current; Adult: 18 to 64 years OR Aged: 65+ years
	**PubMed**: *As per Embase Free Text*	Publication date from 2013/05/01 to 2016/07/04; Humans; English; Adult: 19+ years

**Fig. 1:**
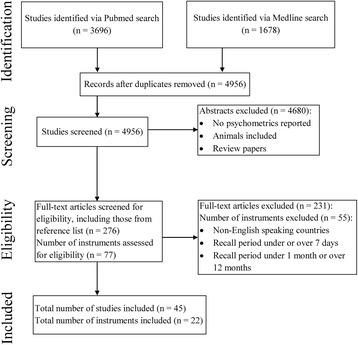
Flowchart of included studies and physical activity questionnaires

From the search strategy, a total of 4056 abstracts were retrieved including duplicates. Duplicates (*n* = 75) were removed and which resulted in 3981 abstracts that underwent further screening. The summary of the search process is presented in Fig. 1.

#### Selection process

Two independent reviewers conducted the stepwise literature search. Firstly, all titles and abstracts that potentially met the eligibility criteria were screened as either meeting the eligibility criteria (“yes”), potentially meeting the eligibility criteria (“maybe”) or not meeting the eligibility criteria (“no”). Following abstract screening, a random sample (40%) of the abstracts was reviewed to determine the inter-rater reliability between both reviewers. A Weighted Kappa calculation of 0.76 (95% CI: 0.71–0.82) was obtained and considered as acceptable for inter-rater reliability [23]. Following this confirmation, all corresponding original journal articles (both “yes” and “maybe”) were retrieved and further screening was undertaken based on the inclusion/exclusion criteria.

### Methodological quality using COSMIN taxonomy

The methodological quality of included studies was assessed using the COSMIN taxonomy of measurement properties with definitions for health-related patient-reported outcomes shown in Table 2. The COSMIN checklist consists of nine domains: *internal consistency*, *reliability* (test-retest reliability, inter-rater reliability and intra-rate reliability), *measurement error* (absolute measures), *content validity*, *structural validity*, *hypothesis testing*, *cross-cultural validity*, *criterion validity* and *responsiveness* [15]. Of these domains, *responsiveness*, *cross-cultural validity* and *criterion validity* were not assessed for the following reasons: *responsiveness* – determination of the instrument’s sensitivity to changes over time was beyond the scope of the current review; *cross-cultural validity* – questionnaires assessed in languages other than English were excluded during screening; and *criterion validity* – currently, there is no globally-accepted ‘golden standard’ based on consensus for assessing PA level [24, 25]. *Interpretability* was not examined as this component is not considered as a psychometric property. Each domain of the COSMIN checklist was assessed using scales consisting of 5 to 18 items that addressed issues on study design and statistical analyses. To determine the overall methodological quality per domain, [15] suggested to report the lowest item rating within the domain using their 4-point rating system (i.e., excellent, good, fair and poor, respectively). However, as this scoring system does not account for subtle differences in the psychometric qualities of each study, a revised version was implemented as previously described [26]. The raw item scores were transformed into a percentage of rating using the following formula:

$$ Total\  score\  of\  each\  domain = \frac{\left( Total\  score\  obtained - minimum\  score\  possible\right)}{\left( Highest\  score\  possibe - minimum\  score\  possible\right)} \times 100 $$

**Table 2:** Definitions for aspects of domains and measurement properties from the COSMIN checklist by Mokkink et al. (2010)

Reliability
The degree to which the measurement is free from measurement error
Internal consistency The degree of the interrelatedness among the items
Reliability The proportion of the total variance in the measurements which is because of “true” differences among patients
Measurement error The systematic and random error of a patient’s score that is not attributed to true changes in the construct to be measured
Validity The degree to which an HR-PRO instrument measures the construct(s) it purports to measure
Content validity The degree to which the content of an HR-PRO instrument is an adequate reflection of the construct to be measured
Face validity The degree to which an HR-PRO instrument indeed looks as though they are an adequate reflection of the construct to be measured
Construct validity The degree to which the scores of an HR-PRO instrument are consistent with hypotheses based on the assumption that a HR-PRO measure validly measures the construct to be measured
Structural validity The degree to which the scores of an HR-PRO measure are an adequate reflection of the dimensionality of the construct to be measured
Hypotheses testing Item construct validity
Cross-cultural validity The extent to which performance of the items from translated or culturally adapted measures adequately replicates the performance of the items from original versions of the measure
Criterion validity The degree to which the scores of a measure adequately reflect a “gold standard”
Responsiveness The measure’s sensitivity to changes in the construct to be measured over time
Interpretability ^a^ The extent to which qualitative meaning can be derived from a measure’s quantitative scores or score change

^a^Interpretability is not considered a psychometric property

The final rating percentage for each domain was then qualitatively defined using the following categories: Poor = 0–25.0%, Fair = 25.1–50.0%, Good = 50.1–75.0%, Excellent = 75.1–100.0% [26]. Furthermore, all studies were appraised by two raters, independently with differences in ratings resolved via consensus.

### Quality of the psychometric properties

To compare the strength of *reliability* (i.e., test-retest reliability) between Usual-week and Past-week PAQs, we calculated the weighted mean of correlation coefficients (i.e., r-values) using the following formula:

$$ \overline{x} = \frac{{\displaystyle {\sum}_{i=1}^n}{w}_i{x}_i}{{\displaystyle {\sum}_{i=1}^n}{w}_i} $$

Where *w* = r-value of each study and *x* = sample size of each study

The weighted means of the r-values were calculated to account for sample size varying between comparisons within studies or between studies. When the sample size of each comparison was identical, the normal non-weighted r-values were averaged. The mean r-values were also calculated to compare the strength of *convergent validity*between Usual-week and Past-week PAQs and between PAQs compared with direct measures (e.g., accelerometers, pedometers, PA diaries) and PAQs with indirect measures (e.g., maximal oxygen consumption test [VO2max]). The strength of the r-values was interpreted based on Cohen’s classifications in the order of 0.10 as weak, those of 0.30 as moderate, and those of 0.50 as strong in terms of magnitude [27].

We also classified the psychometric quality of each measurement property for each study as either “positive” (+),“conflicting” (±), “indeterminate” (?), “negative” (−) “not reported” (NR) or “not evaluated” (NE) using quality criteria as previously described (Table 3) [17, 28]. For example, if the reported intra-class correlation coefficient (ICC) was 0.9 (≥0.7 classified as acceptable), then the psychometric quality for that particular psychometric property of the study will be classified as “positive”. Conversely, if the reported ICC was 0.6 (not acceptable given that it is less than 0.7), then the psychometric quality of the study will be classified as “negative”. If a number of reliability analyses had ICC values of above (i.e., ≥ 0.7) and below (i.e., < 0.7) acceptable standards within the same study, than the psychometric quality of the study will be classified as “conflicting”. Studies that received a poor COSMIN rating were excluded from further analysis and were classified as “not evaluated” (NE).

**Table 3:** Modified criteria of psychometric quality rating based on Terwee, Bot [15] and Cordier, Chen [26]

Psychometric property	Score^a^	Quality Criteria^b^
Content validity	+	A clear description is provided of the measurement aim, the target population, the concepts that are being measured, and the item selection AND target population and (investigators OR experts) were involved in item selection
	?	A clear description of above-mentioned aspects is lacking OR only target population involved OR doubtful design or method
	-	No target population involvement
	±	Conflicting results
	NR	No information found on target population involvement
	NE	Not evaluated due to “poor” methodological quality
Structural validity	+	Factor analysis performed with adequate sample size. Factors should explain at least 50% of the variance
	?	No factor analysis performed and explained variance not mentioned
	-	Factors explain <50% of the variance
	±	Conflicting results
	NR	No information found on structural validity
	NE	Not evaluated due to “poor” methodological quality
Hypothesis testing	+	Specific hypotheses were formulated AND at least 75% of the results are in accordance with these hypotheses; Convergent validity: correlation between similar assessments is at a statistically significant level (*p* < 0.05) and strength of relationship is ≥0.5 which is consistent with the hypothesis; Discriminant validity: uses appropriate statistical analysis (e.g., *t*-test *p* < 0.05 or Cohen’s d effect size ≥0.5)
	?	Doubtful design or method (e.g., no hypotheses)
	-	Less than 75% of hypotheses were confirmed, despite adequate design and methods; Convergent validity: correlation between similar assessments is not at a statistically significant level (*p* ≥ 0.05) and strength of relationship is <0.5 which is inconsistent with hypothesis
	±	Conflicting results between studies within the instrument
	NR	No information found on hypotheses testing
	NE	Not evaluated due to “poor” methodological quality
Internal consistency	+	Factor analyses performed on adequate sample size (7 * # items and 100) AND Cronbach’s alpha(s) calculated per dimension AND Cronbach’s alpha(s) between 0.70 and 0.95
	?	No factor analysis OR doubtful design or method
	-	Cronbach’s alpha(s) <0.70 or >0.95, despite adequate design and method
	±	Conflicting results
	NR	No information found on internal consistency
	NE	Not evaluated due to “poor” methodological quality
Reliability	+	ICC or weighted Kappa 0.70
	?	Doubtful design or method (e.g., time interval not mentioned)
	-	ICC or weighted Kappa <0.70, despite adequate design and method
	±	Conflicting results
	NR	No information found on reliability
	NE	Not evaluated due to “poor” methodological quality
Measurement error^c^	+	MIC < SDC OR MIC outside the LOA OR convincing arguments that agreement is acceptable
	?	Doubtful design or method OR (MIC not defined AND no convincing arguments that agreement is acceptable)
	-	MIC SDC OR MIC equals or inside LOA, despite adequate design and method
	±	Conflicting results
	NR	No information found on measurement error
	NE	Not evaluated due to “poor” methodological quality

^a^Scores: positive rating (+), indeterminate rating (?), negative rating (−), conflicting data (±), not reported (NR), not evaluated (NE)

^b^Doubtful design or method is assigned when a clear description of the design or methods of the study is lacking, sample size smaller than 50 subjects (should be at least 50 in every subgroup analysis), or any important methodological weakness in the design or execution of the study

^c^Measurement error: *MIC* minimal important change, *SDC* smallest detectable change, *LOA* limits of agreement

To determine the overall quality per psychometric property for each PAQ, the methodological quality based on the COSMIN checklist and the psychometric quality based on [17] of each study were combined to determine the Level of Evidence [18], thus generating an overall psychometric quality rating.

### Data items and synthesis of results

Relevant items from the COSMIN checklist and from the quality criteria by [17] and [18] were analysed for each included study. Results were assessed and reported using the following sequence: 1) the description of the systematic literature search; 2) the characteristics of the instruments and description of all studies included in this review; 3) the methodological quality of each study reporting on psychometric properties of included PAQs based on the COSMIN checklist; 4) the psychometric quality based on the criterion by [17] for each psychometric property per study, including a comparison of the magnitude of weighted r-values of *test-retest reliability* and *convergent validity*; 5) the overall rating of psychometric properties using the Levels of Evidence by [18] for each PAQ and its comparison between Usual-week and Past-week PAQs.

## Results

### Systematic literature search

A total of 3981 abstracts were screened based on the inclusion criteria after removal of duplicate abstracts from the two databases. Following screening, 255 original articles and their corresponding 76 PAQs were assessed for eligibility. Of these, 21 PAQs met the inclusion criteria, while 55 PAQs were excluded. Reasons for exclusion of PAQs included: recall period of only 24 h; single-item PAQs; no specific recall periods; recall periods of over 7 days; recall periods of less than 7 days; and a combination of various recall periods. Accordingly, the psychometric properties of 21 PAQs were evaluated using 44 of the corresponding original articles.

### Included physical activity questionnaires

The characteristics of the 21 included PAQs and description of studies for the development and validation of PAQs are displayed in Tables 4 and 5, respectively. Seven PAQs assessed 7-days of Usual PA level with a 12-month recall period for three PAQs, a 3-month recall period for three PAQs, and a 1-month recall period for one PAQ. Conversely, 14 PAQs assessed PA level over the Past 7-days. The subscales for the majority of PAQs were separated by intensity of PA level (e.g., light, moderate and vigorous) although a number of other PAQs were categorised according to mode of activity (e.g., walking, stairs, transportation, occupational and yard activities).

**Table 4:** Characteristics of instruments assessing level of physical activity

Instrument	Purpose of instrument	Reference	Publication year	Type of administration/Recall method	Relevant number of subscales/forms	Total relevant number of items	Response options
EPAQ2 *Usual 7-days*	To assess average weekly physical activity over the past year in home, work and recreational settings	Wareham, Jakes [29]	2002	Usual 7-days over last 12 months	3	72	Type of activity apart from work: • Distance; Hours/day; Frequency/day Type of activity at work: • Hours/week; Frequency/day; ≥ 1 h/day; Distance Recreation • Frequency/week or month; Hours: Minutes/activity
EPIC PAQ *Usual 7-days*	To estimate usual daily energy expenditure over the course of the past year	Pols, Peeters [31]	1997	Usual 7-days over 12 months	4	13	Occupation: • Sedentary; standing; manual; heavy manual Type of activity: • Hours/week Vigorousness of activity: • Yes or No Flights of stairs: • Floors/day
IPEC-WA *Usual 7-days*	To assess frequency and duration of several levels of incidental and planned physical activity in older people during the past 3 months	Delbaere, Hauer [11]	2010	Usual 7-days over 3 months	3	11	Type of activity: • Frequency/week; 0–4 h/session Walking activities: • Frequency/week; 0–4 h/day Household activities: • 0–4 h/day
NHS II *Usual 7-days*	Prospective study of determinants of breast cancer and other major illnesses in young women	Belanger, Speizer [32]	1976	Usual 7-days over 12 months	3	16	Daily flights of stairs: • ≤2; 3–4; 5–9; 10–14; ≥ 15 Physical activity per week: • 0–11+ hours Sedentary time per week: • 0–90+ hours
Seven-day Adventists and non-Adventists (SDANA) *Usual 7-days*	To assess physical activity levels in Adventists	Singh, Tonstad [33]	1996	Usual 7-days over 3 months	4	28	Moderate activity: Standing: Sitting: Sleeping or reclining: • None; 1–14 min; 15–30 min; 30–60 min; 1–2 h; 2–4 h; > 4 h
Stanford Usual Activity Survey *Usual 7-days*	To assess frequency of every-day and recreational physical activity levels	Sallis, Haskell [34]	1985	Usual 7-days over 3 months	2	11	Everyday activities: Recreational activities: • Yes or No
YPAS *Usual 7-days*	To assess physical activity among older adults	Dipietro, Caspersen [35]	1988	Usual 7-days over 1 month	6	39	Type of activity: • Hours/week Vigorousness of activity: • Frequency/week or/month Leisurely walk: • Frequency/week or/month; Duration in minutes General movement: • Hours/day Standing and sitting: • Hours/day Seasonal changes: • Compare current season
AAS *Past 7-days*	Population surveillance of physical activity in Australian adults	Commission [36]	1997	Past 7-days	4	8	Walking activities: Vigorous yard work: Vigorous activities other than yard work: Moderate activities: • Frequency/week; Hours: minutes/week
CAQ-PAI *Past 7-days*	To measure overall kilocalories expended in leisure-time physical activity	Paffenbarger, Wing [37]	1978	Past 7-days	3	4	Walking: • Blocks/day Stairs: • Flights/day Recreational activities: • Frequency/week; Hours: minutes/session
Checklist Questionnaire *Past 7-days*	Assess the frequency and duration of physical activities performed in the previous 7 days	Masse, Fulton [38]	2012	Past 7-days	10	64	*Self-report* Household activities: Yard activities: Family activities: Community/volunteer/church: Transportation: Miscellaneous: Other time: • Frequency/week; Hours: minutes/week *Interview* Exercise, sports and dancing: Employment: Miscellaneous: • Frequency/week; Hours: minutes/week
GPPAQ *Past 7-days*	Assesses the duration of physical activities performed in the previous 7 days	Health [39]	2013	Past 7-days	2	8	Occupational activities General exercise Cycling Walking Housework/Childcare Gardening/DIY • Hours/week
IPAQ-LF *Past 7-days*	Assesses physical activity level that can be used to obtain internationally comparable data.	Tudor-Locke, Ainsworth [40]	2002	Past 7-days	As for IPAQ-LF (Telephone)	As for IPAQ-LF (Telephone)	As IPAQ-LF (Telephone)
IPAQ-SF *Past 7-days*	As for IPAQ-LF	Tudor-Locke, Ainsworth [40]	2002	Past 7-days	4	7	Vigorous activities Moderate activities Light activities Sitting time • Days/week; Hours: minutes/day
IPAQ-SF w/recall confidence *Past 7-days*	To measure self-reported confidence ratings in recall of physical activity based on IPAQ-SF (self-administered)	Cust, Armstrong [41]	2009	Past 7-days	5	14	Vigorous activities: Moderate activities: Light activities: Sedentary activities: • Days/week; Hours: minutes/day Confidence ratings: • Very unsure; quite unsure; about 50/50; quite sure; very sure
IPEC-WA *Usual 7-days*	To assess frequency and duration of several levels of incidental and planned physical activity in older people during the past 3 months	Delbaere, Hauer [11]	2010	Past 7-days	3	11	Type of activity: • Frequency/week; 0–4 h/session Walking activities: • Frequency/week; 0–4 h/day Household activities: • 0–4 h/day
OSPAQ *Past 7-days*	To measure occupational sitting, standing, and physical activity time	Chau, Van Der Ploeg [42]	2012	Past 7-days	2	6	Time at occupation: • Hours/week; Days/week Occupational activities: • Percentage of total time at work
OSWEQ *Past 7-days*	To monitor PA via the Web	Taylor, Lawton [43]	2013	Past 7-days	-	-	-
Physical Activity Scale for the Elderly (PASE) *Past 7-days*	To assess leisure, occupational and household physical activities amongst the elderly	Washburn, Smith [44]	1991	Past 7-days	3	27	Recreational activities: • Frequency/week; < 1 h, 1–2 h, 2–4 h or >4 h Household activities: • Yes or no; Type of activities Occupational activities: • Hours/week; Type of activities
PA Recall Instrument *Past 7-days*	Assess multiple domains of physical activity against accelerometer data among overweight and non-overweight adults	Timperio, Salmon [45]	2003	Past 7-days	3	3	Light activities: Moderate activities: Vigorous activities: • Frequency/week for ≥10 min
SPAQ2 *Past 7-days*	To measure stage of exercise behaviour change and 7 day recall of physical activity	Lowther, Mutrie [46]	1997	Past 7-days	2	8	Recreational activities: Occupational activities: • Hours/day; Hours/week
Stanford 7-day Physical Activity Recall (PAR) *Past 7-days*	To assess sleep and physical activity patterns	Sallis, Haskell [34]	1985	Past 7-days	6	15	Occupational activities: • Yes or No; Frequency/week; Hours/week; Days/week Moderate, Hard and Very Hard in the Morning: Moderate, Hard and Very Hard in the Afternoon: Moderate, Hard and Very Hard in the Evening: Strength: Flexibility: • Minutes
TPAQ *Past 7-days*	To measures physical activities for recreation and transport	Adams, Goad [47]	2014	Past 7-days	3	21	Transport to work; business; school; shops; friends: • Frequency/week; Hours: minutes/travel; Distance/travel (miles) Recreational activities for walking and cycling: • Frequency/week; Hours: minutes/week Vigorous and moderate-vigorous activities: • Frequency/week • Hours: minutes/week

*EPAQ2* EPIC Physical Activity Questionnaire 2, *EPIC PAQ* EPIC Physical Activity Questionnaire, *IPEC-WA* Incidental and Planned Exercise Questionnaire for the Usual-week, *IPEC-W* Incidental and Planned Exercise Questionnaire for the Past-week, *NHS II* Nurse’s Health Study, *SDANA* Seven-day Adventists and non-Adventists, *YPAS* Yale Physical Activity Survey, *AAS* Active Australia Survey, *CAQ-PAI* College Alumnus Questionnaire Physical Activity Index, *GPPAQ* General practice physical activity questionnaire, *IPAQ-LF* International Physical Activity Questionnaire – Long Form, *IPAQ-SF* International Physical Activity Questionnaire – Short Form, *OSPAQ* Occupational Sitting & Physical Activity Questionnaire, *OSWEQ* Online Self-reported Walking and Exercise Questionnaire, *PASE* Physical Activity Scale for the Elderly, *SPAQ2* Scottish Physical Activity Questionnaire, *PAR* Stanford 7-day Physical Activity Recall, *TPAQ* Transport Physical Activity Questionnaire

**Table 5:** Description of studies for the development and validation of usual-week and past-week physical activity questionnaires

Instrument	Reference	Purpose of study	Study population	Age range (R; mean ± standard deviation) or (R; median [IQR])
EPAQ2*Usual 7-days*	Espana-Romero, Golubic [48]	To compare physical activity sub-components of the EPIC PAQ with a combined heart rate and body movement sensor	*Male (I) & Female (II)*: Validity (*N* = 813 & 876)	*Total sample*: *R* = NR (NR); *(I)* NR (62.8 ± 1.2)y; *(II)* NR (62.9 ± 1.1)y
	Golubic, Martin [49]	Validity of EPAQ2 with accelerometers amongst early old-aged adults	*Combined unemployed/employed (I) & Unemployed (II)*: Validity (*N* = 1705 * 819)	*Total sample*: 60–64 (NR); *(I)* NR; *(II)* NR
	Wareham, Jakes [29]	1) To compare EPAQ2 with measures of energy expenditure assessed by heart rate monitoring; 2) to assess the repeatability of the questionnaire	*Male*: Repeatability *(I)* & Validity *(II)* (*N* = 187 & 84) *Female*: Repeatability *(III)* & Validity *(IV)* (*N* = 212 & 89)	*Total sample*: *R* = NR (64.6 ± 8.4); *(I)* NR (65.0 ± 8.2)y; *(II)* NR (58.8 ± 7.9)y; *(III)* NR (63.8 ± 8.4)y; *(IV)* NR (55.4 ± 6.7)y
EPIC PAQ *Usual 7-days*	Cust, Smith [27]	To examine the validity and long-term repeatability of total physical activity measurements estimated from the past-year recall EPIC questionnaire, using accelerometers as an objective reference measure	Validity & Repeatability (*N* = 182)	*Total sample*: *R* = 50–65 (NR)y
	Cust, Armstrong [41]	To assess whether self-reported confidence in recall of physical activity was a predictor of the validity and retest reliability of the EPIC PAQ and IPAQ	Validity & Repeatability (*N* = 177)	*Total sample*: *R* = 50–65 (NR)y
	Wareham, Jakes [50]	1) Compared the EPIC PAQ with energy expenditure assessed by heart rate monitoring with individual calibration; and; 2) Assessed the repeatability of the EPIC PAQ	Validity (*N* = 173)	*Total sample*: *R* = NR (57.1 ± 7.3)y
IPEQ-W *Usual 7-days*	Delbaere, Hauer [11]	As for IPEQ-WA	As for IPEQ-WA	As for IPEQ-WA
IPEQ-WA *Usual 7-days*	Delbaere, Hauer [11]	Assess the validity and reliability of the IPEQ-W and IPEQ-WA instruments	*IPEQ-W*: Repeatability *(I)* & Validity *(II)* (*N* = 50 & 115) *IPEQ-WA*: Repeatability *(III)* & Validity *(IV)* (*N* = 30 & 106)	*Total sample*: *R* = NR (77.4 ± 6.1)y; *(I)* NR; *(II)* NR; *(III)* NR; *(IV)* NR
NHS II*Usual 7-days*	Wolf, Hunter [51]	Reproducibility and validity of the NHS II PAQ and 2 physical inactivity questions	*Non-African-American (I) & African-American (II)*: Repeatability (*N* = 153 & 96); Validity (*N* = 169 & 105)	*Total sample*: *R* = 25–42 (39.0 ± 4.6)y; *(I) R* = NR (39 ± 4.3)y; *(II) R* = NR (39 ± 4.5)y
Seven-day Adventists and non-Adventists (SDANA) *Usual 7-days*	Singh, Tonstad [33]	Construct validity and reliability of white Seventh-day Adventists and non-Adventists physical activity questionnaire	*Adventist males (I), Non-Adventist males (II), Adventist females (III) & Non-Adventist females*: Validity (*N* = 55, 59, 56 & 34), Repeatability (*N* = 28, 31, 41 & 12)	*Total sample*: NR; *(I)* NR (53.6 ± 15.2)y; *(II)* NR (50.0 ± 11.9)y; *(III)* NR (54.6 ± 17.0)y; *(IV)* NR (50.4 ± 11.8)y *Note.* Not separated by ‘validity’ and ‘repeatability’
	Singh, Fraser [52]	Convergent validity and reliability of white Seventh-day Adventists and non-Adventists physical activity questionnaire	Validity *(I)* (*N* = 104), Repeatability *(II)* (*N* = 138)	*Total sample*: NR (49 ± 14.5)y; *(I)* NR; *(II)* NR
Stanford Usual Activity Questionnaire *Usual 7-days*	Jacobs, Ainsworth [53]	As for CAQ-PAI	As for CAQ-PAI	As for CAQ-PAI
YPAS *Usual 7 days*	Resnicow, McCarty [54]	Convergent validity of physical activity questionnaires with physical fitness	*Total sample (I), Male (II), Female (III), Income (IV) & Education (V)*: Validity (*N* = 138, 29, 109, 122 & 138)	*(I) Total sample*: *R* = 21–68 (40.7 ± 8.9)y; *(II)* NR; *(III)* NR, *(IV)* NR, *(V)* NR
AAS *Past 7-days*	Brown, Burton [55]	Assessed the test-retest reliability (repeatability) and validity of the modified version of the Active Australia survey as used in the Australian Longitudinal Study of Women’s Health	Validity *(I)* (*N* = 44); Repeatability *(II)* (*N* = 159)	*Total sample*: *R* = NR (54.9 ± 1.4)y; *(I)* NR (54.7 ± 1.6)y; NR (54.9 ± 1.4)y
CAQ-PAI *Past 7-days*	Ainsworth, Berry [56]	Construct validity of CAQ-PAI with physical fitness	*Inactive (I), Low active (II) & Active (III)*: Validity (*N* = 37, 99 & 53)	*Total sample*: NR (18.3 ± 1.0)y, *(I)* NR, *(II)* NR, *(III)* NR
	Ainsworth, Leon [57]	Measured test-retest reliability and convergent validity of CAQ-PAI	*Male (I) & Female (II)*: Validity & Repeatability (*N* = 78)	*Total sample*: 21–59 (38.0 ± 9.0)y; *(I)* NR; *(II)* NR
	Albanes, Conway [58]	Convergent validity between eight physical activity questionnaires	Validity (*N* = 21)	*Total sample*: 28–55 (37.8 ± 1.8)y
	Bassett, Cureton [59]	Convergent validity of CAQ-PAI with pedometers	*Combined gender (I), Male (II) & Female (III)*: Validity (*N* = 96, 48 & 48)	*(I) Total sample*: 25–70 (39.9 ± 11.3)y, *(II)* NR (40.9 ± 11.2)y, *(III)* NR (39.0 ± 11.5)y
	Jacobs, Ainsworth [53]	Compared physical activity questionnaires with objective and subjective measures of physical activity	Validity & Repeatability (*N* = 78)	*Total sample*: 20–59 (37.3 ± 9.9)y
	Resnicow, McCarty [54]	As for YPAS	As for YPAS	As for YPAS
	Strath, Bassett [60]	Compared estimates of intensity-specific and total physical activity measured by CAQ-PAI to those measured by the heart rate and motion sensor, over 7 days	*Male (I), Female (II)*: Validity (*N* = 12 & 13)	*Total sample*: 20–56 (30.0 ± 10.5)y; *(I)* NR (30.6 ± 9.9)y; *(II)* NR (29.5 ± 11.4)y
	Washburn, Goldfield [61]	Construct validity of self-reported activities enough to induce sweating	*Combined gender (I), Male (II), Female (III), age 25–39 (IV) & age 40–65 (V)*: Validity (*N* = 657, 275, 382, 375 & 282)	*(I) Total sample*: 25–65 (39.5 ± 10.8)y; *(II)* NR (38.2 ± 10.6); *(III)* NR (40.5 ± 10.8)y; *(IV)* NR (31.7 ± 4.3)y; *(V)* NR (49.8 ± 7.6)y
Checklist Questionnaire *Past 7-days*	Masse, Fulton [38]	Compared the validity of two physical activity questionnaire formats	Validity (*N* = 260)	*Total sample*: *R* = 40–70 (49.2 ± 7.0)y
GPPAQ	Ahmad, Harris [62]	Assess reliability and validity of GPPAQ in the elderly	Validity (*N* = 298) Repeatability (*N* = 148)	*Total sample: R* = 60–74 (NR)
IPAQ-LF (self-administered) *Past 7-days*	McKeon, Slevin [63]	Pilot study to validate the Sensewear Armband and the International Physical Activity Questionnaire in developing a methodology to measure and explore the physical activity of men with intellectual disability	Validity (*N* = 17)	*Total sample*: *R* = 19–59 (NR)
IPAQ-SF (Self-administered) *Past 7-days*	Cust, Armstrong [41]	As for EPIC PAQ	As for EPIC PAQ	As for EPIC PAQ
	Kaleth, Ang [64]	To determine the construct validity and test–retest reliability of two self-report physical activity questionnaires IPAQ-SF and CHAMPS in a fibromyalgia population	Validity & Repeatability (*N* = 30)	*Total sample*: *R* = 28–72 (49.1 ± 9.6)y
	Tierney, Fraser [65]	Convergent validity of IPAQ-SF (telephone) with Sense Wear Armband amongst patients with rheumatoid arthritis	Validity (*N* = 22)	*Total sample*: NR (60 ± 13)y
	Warner, Wolin [66]	To examine whether agreement between self-reported and accelerometer measured physical activity varies by BMI category in a low-income black sample	*Obese (I) & Non-obese (II)*: Validity (*N* = 74 & 61)	*Total sample*: *R* = 24–64 (43.4 ± 11.6)y; *(I) R* = NR (42.9 ± 12.1)y; *(II) R* = NR (43.9 ± 11.0)y
IPAQ-SF (recall confidence) *Past 7-days*	Cust, Armstrong [41]	As for EPIC PAQ	As for EPIC PAQ	As for EPIC PAQ
OSPAQ *Past 7-days*	Chau, Van Der Ploeg [42]	Developed and validated OSPAQ	Validity (*N* = 85); Repeatability (*N* = 84)	*Total sample*: 19- ≥ 60 (NR)y
	Chau, Van Der Ploeg [42]	Validity of the OSPAQ with accelerometers	*Combined waist/thigh (I), waist (II) & thigh (III)*: Validity (*N* = 41, 22, 19)	*Total sample (I)*: 18–50+ (NR)y; *(II)*: NR; *(III)* NR
OSWEQ *Past 7-days*	Taylor, Lawton [43]	To 1) develop an online PA questionnaire for estimating energy expenditure (EE) and time spent in moderate-to-vigorous physical activity (MVPA); 2) examine the test-retest reliability of the new online PA questionnaire and the IPAQ short form; and 3) compare the online questionnaire, IPAQ short form, and accelerometer measurement for EE and time spent in MVPA	Validity *(I)* (*N* = 49); Repeatability *(II)* (*N* = 59)	*Total sample*: NR (27 ± 11.9)y; *(I)* NR; *(II)* NR (27 ± 11.9)y
PASE *Past 7-days*	Allison, Keller [67]	Reliability and validity of PASE amongst elderly patients in a rural community	Validity and Repeatability (*N* = 32)	*Total sample*: 67–83 (72 ± 4.24)y
	DePew, Garofoli [28]	Convergent validity of PASE with accelerometers amongst patients with chronic obstructive pulmonary disease	Validity and Repeatability (*N* = 67)	*Total sample*: NR (71.4 ± 7.91)
	Ewald, McEvoy [68]	Convergent validity of PASE with pedometers in older adults	*Male (I) & Female (II)*: Validity (*N* = 319 & 350)	*Total sample*: 55–85 (66.3 ± 7.7)y; *(I)* NR, *(II)* NR
	Garfield, Canavan [69]	Convergent validity of three physical activity questionnaires with accelerometers	Validity (*N* = 43)	*Total sample*: NR (68.0 ± 9.0)y
	Granger, Parry [70]	Convergent validity of EPIC with accelerometers amongst patients with lung cancer	Validity (*N* = 69)	*Total sample*: NR (68.0 [61.5–74.0]y
	Harada, Chiu [71]	Assess the known-groups and construct validity of CHAMPS, PASE and YPAS	*Retirement homes (I) & Community centres (II)*: Validity (*N* = 36 & 51)	*Total sample*: *R* = 56–89 (75.0 ± 6.0); *(I) R* = 65–89 (79.0 ± 6.0); *(II) R* = 65–86 (73.0 ± 5.0)
	Martin, Rejeski [72]	Convergent validity of PASE with physiological measures in physical disability	Validity (*N* = 471)	*Total sample*: 65+ (71.7 ± 4.9)y
	Washburn, Smith [44]	Convergent validity and reliability of PASE with accelerometers	Validity & Repeatability (*N* = 119)	*Total sample*: NR (73.4 ± NR)y
	Washburn and Ficker [73]	Convergent validity of PASE with accelerometers	*Total sample (I), age ≤70y (II) & age >70y (III)*: Validity (*N* = 20, 9, 11)	*(I) Total sample*: NR; *(II)* NR; *(III)* NR
	Washburn, McAuley [74]	Construct validity of PASE with physiological measures	*Total sample (I), Male (II), Female (III), age 55–64y (IV), age ≥65y (V)*: Validity (*N* = 190, 56, 134, 87 & 102)	*(I) Total sample*: 55–65+ (NR)y; *(II)* NR; *(III)* NR; *(IV)* NR; *(V)* NR
	Zalewski, Smith [75]	Convergent validity of PASE with accelerometers in older adults	Validity (*N* = 590	*Total sample*: NR (83.8 ± NR)y
PA Recall Instrument *Past 7-days*	Timperio, Salmon [45]	Validity and reliability of physical activity recall instrument among overweight and non-overweight men and women	*Male total sample (I), Male BMI ≤25 (II), Male BMI >25 (III), Female total sample (IV), Female BMI ≤25 (V), Female BMI >25 (VI)*: Reliability (*N* = 55, 28, 27, 63, 40 & 23), Validity (*N* = 57, 28, 29, 59, 36 & 23)	*Total sample*: 18+ (38.7 ± 14.9)y, *(I)* 18+ (37.8 ± 12.7)y, *(II)* NR, *(III)* NR, *(IV)* 18+ (39.6 ± 17.0)y, *(V)* NR, *(VI)* NR
SPAQ2 *Past 7-days*	Lowther, Mutrie [46]	To establish the test-retest reliability and convergent validity of the SPAQ2	Validity *(I)* (*N* = 96); Repeatability *(II)* (*N* = 34)	*Total sample*: NR; *(I)* NR (33.0 ± 11.6)y; NR (33.0 ± 11.5)y
Stanford 7-day Physical Activity Recall (PAR) *Past 7-days*	Ainsworth, Jacobs [76]	Validity and reliability of occupational physical activity from PAR	Validity & Repeatability (*N* = 75)	*Total sample*: NR (37.3 ± 9.5)y
	Ainsworth, Richardson [77]	Convergent validity of occupational activity between physical activity questionnaires	Validity (*N* = 46)	*Total sample*: 20–60 (39.4 ± 11.8)y
	Dishman and Steinhardt [78]	Reliability and convergent validity of PAR in college students	*(I) Study 1*: Validity & Reliability (*N* = 158), *(II) Study 2*: Validity (*N* = 91) *(III), Study 3*: Validity (*N* = 74), *(IV) Study 4*: Validity (*N* = 24)	*Total sample*: NR (21.9 ± 2.9)y; *(I)* NR, *(II)* NR *(III)* NR, *(IV)* NR (23.0 ± 3.0)y
	Jacobs, Ainsworth [53]	As for CAQ-PAI	As for CAQ-PAI	As for CAQ-PAI
TPAQ *Past 7-days*	Adams, Goad [47]	Reliability and convergent validity of TPAQ with accelerometers	*Moderate intensity (I), Moderate-vigorous intensity (II) & Vigorous intensity (III)*: Validity (*N* = 53, 52 & 46) *Walking for transport (I), Cycling for transport (II), Walking for recreation (III), Cycling for recreation (IV), Moderate intensity (V), Vigorous intensity (VI) & Total physical activity (VII)*: Repeatability (*N* = 164, 164, 165, 165, 165, 163 & 161)	*Total sample for validity*: NR *Total sample for reliability* : NR

*EPAQ2* EPIC Physical Activity Questionnaire 2, *EPIC PAQ* EPIC Physical Activity Questionnaire, *IPEC-WA* Incidental and Planned Exercise Questionnaire for the Usual-week, *IPEC-W* Incidental and Planned Exercise Questionnaire for the Past-week, *NHS II* Nurse’s Health Study, *SDANA* Seven-day Adventists and non-Adventists, *YPAS* Yale Physical Activity Survey, *AAS* Active Australia Survey, *CAQ-PAI* College Alumnus Questionnaire Physical Activity Index, *GPPAQ* General practice physical activity questionnaire, *IPAQ-LF* International Physical Activity Questionnaire – Long Form, *IPAQ-SF* International Physical Activity Questionnaire – Short Form, *OSPAQ* Occupational Sitting & Physical Activity Questionnaire, *OSWEQ* Online Self-reported Walking and Exercise Questionnaire, *PASE* Physical Activity Scale for the Elderly, *SPAQ2* Scottish Physical Activity Questionnaire, *PAR* Stanford 7-day Physical Activity Recall, *TPAQ* Transport Physical Activity Questionnaire

### Psychometric properties of PAQs

Based on the COSMIN rating method for all included 21 PAQs (Table 6), none of the studies showed “poor” ratings and thus the psychometric qualities of all studies were rated. The most frequently reported psychometric properties were *hypothesis testing* (all 21 PAQs) which ranged from good to excellent quality. This was followed by reliability testing (18 PAQs), which ranged from fair to excellent quality; *content validity* (7 PAQs), which ranged from fair to excellent quality; and *internal consistency* (6 PAQs), which ranged from fair to excellent quality. The least reported psychometric properties were *structural validity* (2 PAQs) with good qualities and *measurement error* (2 PAQs) ranging from good to excellent quality.

**Table 6:** Overview of the methodological quality assessment of usual-week and past-week physical activity questionnaires using the COSMIN checklist

Instrument	Study	Measurement properties					
		Reliability			Content validity	Construct Validity	
		Internal consistency	Reliability testing^a^	Measurement error		Structural validity	Hypothesis testing^bc^
EPAQ2 *Usual 7-days*	Espana-Romero, Golubic [48]	NR	NR	NR	NR	NR	Direct: 80.6% (Excellent) Discriminant: 78.1% (Excellent)
	Golubic, Martin [49]	NR	NR	NR	NR	NR	Direct: 81.3% (Excellent) Discriminant: 83.3% (Excellent)
	Wareham, Jakes [29]	NR	86.1% (Excellent)	NR	65.0% (Good)	NR	Direct: 77.8% (Excellent) Indirect: 77.8%(Excellent)
EPIC PAQ *Usual 7-days*	Cust, Smith [27]	NR	77.3% (Excellent)	81.8% (Excellent)	NR	NR	Direct: 80.6% (Excellent) Discriminant: 87.5% (Excellent)
	Cust, Armstrong [41]	NR	77.3% (Excellent)	NR	NR	NR	Direct: 77.8% (Excellent)
	Wareham, Jakes [50]	NR	76.9% (Excellent)	NR	70.0% (Good)	NR	Direct: 83.3% (Excellent) Indirect: 80.6% (Excellent) Discriminant: 81.3% (Excellent)
IPEQ-WA *Usual 7-days*	Delbaere, Hauer [11]	84.4 (Excellent)	75.0% (Good)	NR	NR	75.0% (Good)	Direct: 75.0% (Good)
NHS II *Usual 7-days*	Wolf, Hunter [51]	NR	73.9% (Good)	NR	NR	NR	Direct: 77.8% (Excellent) Discriminant: 65.6% (Good)
SDANA *Usual 7-days*	Singh, Tonstad [33]	55.0 (Good)	73.1% (Good)	NR	70.0% (Good)	NR	Indirect: 75.0% (Good) Discriminant: 71.9% (Good)
	Singh, Fraser [52]	NR	NR	NR	NR	NR	Direct: 83.3% (Excellent) Indirect: 80.6% (Excellent) Discriminant: 75.0% (Good)
Stanford Usual Activity Questionnaire *Usual 7-days*	Jacobs, Ainsworth [53]	NR	72.7% (Good)	NR	NR	NR	Direct: 72.2% (Good) Indirect: 75.0% (Good)
YPAS *Usual 7 days*	Resnicow, McCarty [54]	NR	NR	NR	NR	NR	Indirect: 77.8% (Excellent)
AAS *Past 7-days*	Brown, Burton [55]	NR	86.4% (Excellent)	NR	NR	NR	Direct: 75.0% (Good) Discriminant: 65.6% (Good)
CAQ-PAI *Past 7-days*	Ainsworth, Berry [56]	NR	NR	NR	NR	NR	Indirect: 83.3% (Excellent)
	Ainsworth, Leon [57]	NR	65.9% (Good)	NR	NR	NR	Direct: 72.2% (Good) Indirect: 69.4% (Good)
	Albanes, Conway [58]	32.5 (Fair)	NR	NR	NR	NR	Direct: 58.3% (Good)
	Bassett, Cureton [59]	NR	NR	NR	NR	NR	Direct: 72.7% (Good)
	Jacobs, Ainsworth [53]	NR	72.7% (Good)	NR	NR	NR	Direct: 72.2% (Good) Indirect: 75.0% (Good)
	Resnicow, McCarty [54]	NR	NR	NR	NR	NR	Indirect: 77.8% (Excellent)
	Strath, Bassett [60]	NR	NR	NR	NR	NR	Direct: 63.9% (Good) Discriminant: 59.4% (Good)
	Washburn, Goldfield [61]	NR	NR	NR	NR	NR	Indirect: 83.3% (Excellent)
Checklist Questionnaire *Past 7-days*	Masse, Fulton [38]	NR	NR	NR	80.0% (Excellent)	NR	Direct: 72.2% (Good)
GPPAQ *Past 7-days*	Ahmad, Harris [62]	NR	85.4% (Excellent)	NR	NR	NR	Discriminant: 62.5% (Good)
IPAQ-LF *Past 7-days*	McKeon, Slevin [63]	NR	NR	NR	NR	NR	Direct: 63.9% (Good)
IPAQ-SF *Past 7-days*	Kaleth, Ang [64]	NR	75.0% (Good)	NR	NR	NR	Direct: 63.9% (Good)
	Tierney, Fraser [65]	NR	NR	NR	NR	NR	Direct: 69.4% (Good)
	Warner, Wolin [66]	NR	NR	NR	NR	NR	Direct: 80.6% (Excellent)
IPAQ-SF (recall confidence) *Past 7-days*	Cust, Armstrong [41]	NR	77.3% (Excellent)	NR	NR	NR	Direct: 77.8% (Excellent)
IPEQ-W *Past 7-days*	Delbaere, Hauer [11]	84.4 (Excellent)	75.0% (Good)	NR	NR	75.0 (Good)	Direct: 75.0% (Good)
OSPAQ *Past 7-days*	Chau, Van Der Ploeg [42]	NR	50.0% (Fair)	NR	NR	NR	Direct: 75.0% (Good)
	Jancey, Tye [79]	NR	79.5% (Excellent)	NR	NR	NR	Direct: 72.2% (Good)
OSWEQ *Past 7-days*	Taylor, Lawton [43]	NR	50.0% (Good)	NR	45.0% (Fair)	NR	Direct: 75.0% (Good)
PASE *Past 7-days*	Allison, Keller [67]	72.2 (Good)	75.0% (Good)	NR	80.0% (Excellent)	NR	NR
	DePew, Garofoli [28]	NR	67.5% (Good)	70.5% (Good)	NR	NR	NR
	Ewald, McEvoy [68]	NR	NR	NR	NR	NR	Direct: 83.3% (Excellent)
	Garfield, Canavan [69]	NR	NR	NR	NR	NR	Direct: 77.8% (Excellent)
	Granger, Parry [70]	NR	NR	NR	NR	NR	Direct: 88.9% (Excellent)
	Harada, Chiu [71]	NR	NR	NR	NR	NR	Direct: 72.2% (Good) Indirect: 69.4% (Good)
	Martin, Rejeski [72]	NR	NR	NR	NR	NR	Indirect: 80.6% (Excellent)
	Washburn, Smith [44]	69.4 (Good)	72.7% (Good)	NR	75.0% (Good)	NR	Indirect: 75.0% (Good)
	Washburn and Ficker [73]	NR	NR	NR	NR	NR	Direct: 66.7% (Good)
	Washburn, McAuley [74]	NR	NR	NR	NR	NR	Indirect: 83.3% (Excellent)
	Zalewski, Smith [75]	NR	NR	NR	NR	NR	Direct: 80.6% (Excellent) Indirect: 80.6% (Excellent)
PA Recall Instrument	Timperio, Salmon [45]	NR	71.5% (Good)	NR	NR	NR	Direct: 75.0% (Good)
SPAQ2 *Past 7-days*	Lowther, Mutrie [46]	NR	45.0% (Fair)	NR	NR	NR	Direct: 72.2% (Good) Discriminant: 75.0% (Good)
Stanford 7-day Physical Activity Recall (PAR) *Past 7-days*	Ainsworth, Jacobs [76]	NR	79.5% (Excellent)	NR	NR	NR	Direct: 80.0% (Excellent)
	Ainsworth, Richardson [77]	NR	NR	NR	NR	NR	Direct: 80.6% (Excellent)
	Dishman and Steinhardt [78]	54.5 (Good)	63.6% (Good)	NR	NR	NR	Direct: 72.2% (Good) Indirect: 72.2% (Good)
	Jacobs, Ainsworth [53]	NR	72.7% (Good)	NR	NR	NR	Direct: 72.2% (Good) Indirect: 75.0% (Good)
TPAQ *Past 7-days*	Adams, Goad [47]	NR	77.1% (Excellent)	NR	70.0% (Good)	NR	Direct: 77.8% (Excellent)

*Notes.* ^a^All test-retest reliability tests; ^b^Direct comparisons of physical activity measures (e.g., physical activity level between PAQ and other PAQs, diaries or objective measures) for convergent validity; ^c^Indirect comparisons of physical activity measures (e.g., physical activity level between PAQ and physical fitness, given the assumption that individuals with greater level of physical activity would have a greater level of physical fitness) for construct validity

*EPAQ2* EPIC Physical Activity Questionnaire 2, *EPIC PAQ* EPIC Physical Activity Questionnaire, *IPEC-WA* Incidental and Planned Exercise Questionnaire for the Usual-week, *IPEC-W* Incidental and Planned Exercise Questionnaire for the Past-week, *NHS II* Nurse’s Health Study, *SDANA* Seven-day Adventists and non-Adventists, *YPAS* Yale Physical Activity Survey, *AAS* Active Australia Survey, *CAQ-PAI* College Alumnus Questionnaire Physical Activity Index, *GPPAQ* General practice physical activity questionnaire, *IPAQ-LF* International Physical Activity Questionnaire – Long Form, *IPAQ-SF* International Physical Activity Questionnaire – Short Form, *OSPAQ* Occupational Sitting & Physical Activity Questionnaire, *OSWEQ* Online Self-reported Walking and Exercise Questionnaire, *PASE* Physical Activity Scale for the Elderly, *SPAQ2* Scottish Physical Activity Questionnaire, *PAR* Stanford 7-day Physical Activity Recall, *TPAQ* Transport Physical Activity Questionnaire

Table 7 provides a comparison of the magnitude of the weighted mean of the r-values for *test-retest reliability* and *convergent validity*. The magnitude of the weighted mean of the r-values of PAQs were compared with direct measures (e.g., other PAQs, diaries or objective measures) or indirect measures (e.g., VO2max test). A further comparison was done between the magnitude of the weighted mean of the r-values for *test-retest reliability* of Usual-week and Past-week PAQs. The magnitude of the r-values for both Usual-week and Past-week PAQs were comparable (*r* = 0.62) with similar sample sizes (*n* = 1071 and 901, respectively). Only one study (Stanford Usual Activity Questionnaire) compared *test-retest reliability* between both direct (accelerometer) and indirect (VO2max test) measures with both objective measures showing higher *test-retest reliability* (*r* = 0.67 and 0.68, respectively) than the Stanford Usual Activity Questionnaire (Subjective measure; *r* = 0.46). When comparing convergent validity between recall methods, the magnitude of the weighted mean of the r-values appeared greater for Past-week than Usual-week, particularly when PAQs were compared against direct measures with a moderately strong relationship for the Past-week (*r* = 0.33) versus a weak relationship for the Usual-week (*r* = 0.20) PAQs. When examining the weighted mean of the r-values between PAQs compared against direct measures and indirect measures, similar results were found for Usual-week PAQs (*r* = 0.20 and 0.13, respectively) and when Usual-week and Past-week PAQs were combined (*r* = 0.25 and 0.22, respectively). However, there was a moderate relationship between Past-week PAQs and direct measures (*r* = 0.33) compared to a weak relationship between Past-week PAQs and indirect measures (*r* = 0.24).

**Table 7:** The weighted mean of the correlation coefficients (r-value) for reliability testing and validity of Past-week and Usual-week PAQs

Reliability Testing		
Instrument	r-values	Sample (*n*)
EPAQ2 *Usual-week*	0.66	399
EPIC-PAQ^K^ *Usual-week*	0.65	270
IPEQ-WA^I^ *Usual-week*	NR	NR
NHS II *Usual-week*	0.51	231
SDANA *Usual-week*	0.69	112
Stanford *Usual-week*	0.46	59
YPAS *Usual-week*	NR	NR
AAS *Past-week*	0.59	159
CAQ-PAI *Past-week*	0.69	118
Checklist *Past-week*	NR	NR
IPAQ-LF *Past-week*	NR	NR
IPAQ-SF^I^ *Past-week*	NR	NR
IPAQ-SF-R *Past-week*	0.46	83
IPEQ-W^I^ *Past-week*	NR	NR
OSPAQ^**I**^ *Past-week*	NR	NR
OSWEQ *Past-week*	0.74	49
PASE *Past-week*	0.75	218
PA Recall^I^ *Past-week*	NR	NR
SPAQ *Past-week*	0.99	34
Stanford *Past-week*	0.48	240
TPAQ^I,K^ *Past-week*	NR	NR
Average for Usual-week PAQs	0.62	1071
Average for Past-week PAQs	0.62	901
Validity testing		
EPAQ2 *Usual-week*		
Direct & Indirect	0.18	4386
Direct	0.18	4386
Indirect	0.03	173
EPIC-PAC *Usual-week*		
Direct & Indirect	0.22	266
Direct	0.21	266
Indirect	NR	NR
IPEQ-WA *Usual-week*		
Direct & Indirect	0.82	50
Direct	0.05	173
Indirect	NR	NR
NHS II *Usual-week*		
Direct & Indirect	0.69	233
Direct	0.69	233
Indirect	NR	NR
SDANA *Usual-week*		
Direct & Indirect	0.20	327
Direct	0.28	138
Indirect	0.16	296
Stanford *Usual-week*		
Direct & Indirect	0.19	69
Direct	0.05	73
Indirect	0.33	64
YPAS *Usual-week*		
Direct & Indirect	0.09	138
Direct	0.43	159
Indirect	0.09	138
AAS *Past-week*		
Direct & Indirect	0.43	159
Direct	0.43	159
Indirect	NR	NR
CAQ-PAI *Past-week*		
Direct & Indirect	0.14	1178
Direct	0.28	297
Indirect	0.12	1064
Checklist *Past-week*		
Direct & Indirect	0.31	220
Direct	0.31	220
Indirect	NR	NR
IPAQ-LF *Past-week*		
Direct & Indirect	NR	NR
Direct	NR	NR
Indirect	NR	NR
IPAQ-SF *Past-week*		
Direct & Indirect	0.41	113
Direct	0.41	113
Indirect	NR	NR
IPAQ-SF-R *Past-week*		
Direct & Indirect	0.27	85
Direct	0.27	85
Indirect	NR	NR
IPEQ-W *Past-week*		
Direct & Indirect	0.82	50
Direct	0.82	50
Indirect	NR	NR
OSPAQ *Past-week*		
Direct & Indirect	0.49	103
Direct	0.49	103
Indirect	NR	NR
OSWEQ *Past-week*		
Direct & Indirect	0.42	49
Direct	0.42	49
Indirect	NR	NR
PASE *Past-week*		
Direct & Indirect	0.32	2477
Direct	0.38	1242
Indirect	0.33	1671
PA Recall *Past-week*		
Direct & Indirect	0.30	178
Direct	0.30	178
Indirect	NR	NR
SPAQ *Past-week*		
Direct & Indirect	0.13	30
Direct	0.13	30
Indirect	NR	NR
Stanford *Past-week*		
Direct & Indirect	0.24	271
Direct	0.23	271
Indirect	0.26	147
TPAQ *Past-week*		
Direct & Indirect	0.72	46
Direct	0.72	46
Indirect	NR	NR
Average for Usual-week PAQs	Direct & Indirect (*r* = 0.20) Direct (*r* = 0.20) Indirect (*r* = 0.13)	Direct & Indirect (*n* = 5592) Direct (*n* = 5269) Indirect (*n* = 671)
Average for Past-week PAQs	Direct & Indirect (*r* = 0.29) Direct (*r* = 0.33) Indirect (*r* = 0.24)	Direct & Indirect (*n* = 4959) Direct (*n* = 2843) Indirect (*n* = 2882)
Past-week and Usual-week PAQs	Direct (*r* = 0.25) Indirect (*r* = 0.22)	Direct (*n* = 8112) Indirect (*n* = 3553)

Direct – direct measures comparisons of physical activity measures (e.g., physical activity level between PAQ and other PAQs, diaries or objective measures) for convergent validity

Indirect - Indirect comparisons of physical activity measures (e.g., physical activity level between PAQ and physical fitness, given the assumption that individuals with greater level of physical activity would have a greater level of physical fitness) for construct validity

NR - did not report r-values

^I^calculated intraclass correlation coefficient for *test-retest reliability*

^K^calculated kappa for *test-retest reliability*

*EPAQ2* EPIC Physical Activity Questionnaire 2, *EPIC PAQ* EPIC Physical Activity Questionnaire, *IPEC-WA* Incidental and Planned Exercise Questionnaire for the Usual-week, *IPEC-W* Incidental and Planned Exercise Questionnaire for the Past-week, *NHS II* Nurse’s Health Study, *SDANA* Seven-day Adventists and non-Adventists, *YPAS* Yale Physical Activity Survey, *AAS* Active Australia Survey, *CAQ-PAI* College Alumnus Questionnaire Physical Activity Index, *GPPAQ* General practice physical activity questionnaire, *IPAQ-LF* International Physical Activity Questionnaire – Long Form, *IPAQ-SF* International Physical Activity Questionnaire – Short Form, *OSPAQ* Occupational Sitting & Physical Activity Questionnaire, *OSWEQ* Online Self-reported Walking and Exercise Questionnaire, *PASE* Physical Activity Scale for the Elderly, *SPAQ2* Scottish Physical Activity Questionnaire, *PAR* Stanford 7-day Physical Activity Recall, *TPAQ* Transport Physical Activity Questionnaire

Table 8 provides the quality of psychometric properties of Usual-week and Past-week PAQs based on the quality criteria set out by [17]. Table 9 summarises the overall rating of psychometric properties for each PAQ using the levels of evidence by [18]. Overall, the majority of psychometric properties showed “moderate negative” to “strong negative” ratings for both Usual-week and Past-week PAQs. Of these, IPEQ-WA, SDANA, IPAQ-LF, IPEQ-W, OSPAQ, OSWEQ, SPAQ2 and TPAQ were PAQs that did not include psychometric properties with “negative” ratings. Both IPEQ-WA and IPEQ-W demonstrated “indeterminate” and “conflicting” ratings for *internal consistency* and *reliability testing*, respectively, with “moderate positive” ratings for *structural validity* and *hypothesis testing*. For SPAQ2, “limited positive” to “moderate positive” ratings were reported for *reliability testing* and *hypothesis testing*, respectively. When compared between different PAQ recall methods, Past-week PAQs had a greater proportion of “limited positive” to “strong positive” ratings (10 out of 36 ratings = 27.8%) than Usual-week PAQs (4 out of 20 ratings = 20.0%). However, Past-week PAQs had a greater proportion of “moderate negative” to “strong negative” ratings (14 out 36 ratings = 38.9%) than Usual-week PAQs (7 out of 20 ratings = 35.0%). Only few studies reported on *internal consistency*, *measurement error* and *structural validity*. When compared between psychometric properties irrespective of PAQ recall methods, *content validity* had the greatest proportion of PAQs with “limited positive” to “strong positive” ratings (5 out of 7 ratings = 71.4%), whereas reliability testing had the greatest proportion of PAQs with “moderate negative” to “strong negative” ratings (10 out of 18 ratings = 55.6%). Overall, only few psychometric properties were reported with a majority of ratings having received ‘negative’ ratings.

**Table 8:** Quality of psychometric properties based on the criteria by Terwee et al. (2007) and Schellingerhout et al. (2011)

Instrument	Study	Measurement properties of questionnaires					
		Reliability			Content validity	Construct Validity	
		Internal Consistency	Reliability testing	Measurement Error		Structural validity	Hypothesis testing
EPAQ2 *Usual 7-days*	Espana-Romero, Golubic [48]	NR	NR	NR	NR	NR	- (Direct) ? (Discriminant)
	Golubic, Martin [49]	NR	NR	NR	NR	NR	- (Direct) + (Discriminant)
	Wareham, Jakes [29]	NR	±	NR	?	NR	- (Direct) - (Indirect)
EPIC PAQ *Usual 7-days*	Cust, Smith [27]	NR	-	?	NR	NR	- (Direct) + (Discriminant)
	Cust, Armstrong [41]	NR	±	NR	NR	NR	- (Direct)
	Wareham, Jakes [50]	NR	-	NR	-	NR	? (Direct) ? (Indirect) + (Discriminant)
IPEQ-WA *Usual 7-days*	Delbaere, Hauer [11]	?	±	NR	NR	+	+ (Direct)
NHS II *Usual 7-days*	Wolf, Hunter [51]	NR	-	NR	NR	NR	+ (Direct) ? (Discriminant)
SDANA *Usual 7-days*	Singh, Tonstad [33]	?	±	NR	+	NR	- (Indirect) + (Discriminant)
	Singh, Fraser [52]	NR	±	NR	NR	NR	± (Direct) - (Indirect) ? (Discriminant)
Stanford Usual Activity Questionnaire *Usual 7-days*	Jacobs, Ainsworth [53]	NR	-	NR	NR	NR	- (Direct) - (Indirect)
YPAS *Usual 7 days*	Resnicow, McCarty [54]	NR	NR	NR	NR	NR	- (Indirect)
AAS *Past 7-days*	Brown, Burton [55]	NR	-	NR	NR	NR	- (Direct) ? (Discriminant)
CAQ-PAI *Past 7-days*	Ainsworth, Berry [56]	NR	NR	NR	NR	NR	? (Direct)
	Ainsworth, Leon [57]	NR	-	NR	NR	NR	± (Direct) ± (Indirect)
	Albanes, Conway [58]	?	NR	NR	NR	NR	- (Direct)
	Bassett, Cureton [59]	NR	NR	NR	NR	NR	- (Direct)
	Jacobs, Ainsworth [53]	NR	-	NR	NR	NR	- (Direct) - (Indirect)
	Resnicow, McCarty [54]	NR	NR	NR	NR	NR	- (Indirect)
	Strath, Bassett [60]	NR	NR	NR	NR	NR	- (Direct) + (Discriminant)
	Washburn, Goldfield [61]	NR	NR	NR	NR	NR	- (Indirect)
Checklist Questionnaire *Past 7-days*	Masse, Fulton [38]	NR	NR	NR	+	NR	- (Direct)
GPPAQ *Past 7-days*	Ahmad, Harris [62]	NR	-	NR	NR	NR	?
IPAQ-LF *Past 7-days*	McKeon, Slevin [63]	NR	NR	NR	NR	NR	? (Direct)
IPAQ-SF *Past 7-days*	Kaleth, Ang [64]	NR	-	NR	NR	NR	- (Direct)
	Tierney, Fraser [65]	NR	NR	NR	NR	NR	- (Direct)
	Warner, Wolin [66]	NR	NR	NR	NR	NR	- (Direct)
IPAQ-SF (recall confidence) *Past 7-days*	Cust, Armstrong [41]	NR	-	NR	NR	NR	- (Direct)
IPEQ-W *Past 7-days*	Delbaere, Hauer [11]	?	±	NR	NR	+	+ (Direct)
OSPAQ *Past 7-days*	Chau, Van Der Ploeg [42]	NR	+	NR	NR	NR	- (Direct)
	Jancey, Tye [79]	NR	+	NR	NR	NR	+ (Direct)
OSWEQ *Past 7-days*	Taylor, Lawton [43]	NR	±	NR	+	NR	± (Direct)
PASE *Past 7-days*	Allison, Keller [67]	+	-	NR	+	NR	NR
	DePew, Garofoli [28]	NR	-	?	NR	NR	NR
	Ewald, McEvoy [68]	NR	NR	NR	NR	NR	- (Direct)
	Garfield, Canavan [69]	NR	NR	NR	NR	NR	+ (Direct)
	Granger, Parry [70]	NR	NR	NR	NR	NR	+ (Direct)
	Harada, Chiu [71]	NR	NR	NR	NR	NR	+ (Direct) + (Indirect)
	Martin, Rejeski [72]	NR	NR	NR	NR	NR	- (Indirect)
	Washburn and Ficker [73]	NR	NR	NR	NR	NR	- (Direct)
	Washburn, McAuley [74]	NR	NR	NR	NR	NR	- (Indirect)
	Washburn, Smith [44]	-	-	NR	+	NR	- (Indirect)
	Zalewski, Smith [75]	NR	NR	NR	NR	NR	- (Direct) - (Indirect)
PA Recall Instrument	Timperio, Salmon [45]	NR	-	NR	NR	NR	- (Direct)
SPAQ2 *Past 7-days*	Lowther, Mutrie [46]	NR	+	NR	NR	NR	+ (Direct) + (Discriminant)
Stanford 7-day Physical Activity Recall (PAR) *Past 7-days*	Ainsworth, Jacobs [76]	NR	-	NR	NR	NR	- (Direct)
	Ainsworth, Richardson [77]	NR	NR	NR	NR	NR	- (Direct)
	Dishman and Steinhardt [78]	+	-	NR	NR	NR	+ (Direct) - (Indirect)
	Jacobs, Ainsworth [53]	NR	-	NR	NR	NR	- (Direct) - (Indirect)
TPAQ *Past 7-days*	Adams, Goad [47]	NR	±	NR	+	NR	± (Direct)

*EPAQ2* EPIC Physical Activity Questionnaire 2, *EPIC PAQ* EPIC Physical Activity Questionnaire, *IPEC-WA* Incidental and Planned Exercise Questionnaire for the Usual-week, *IPEC-W* Incidental and Planned Exercise Questionnaire for the Past-week, *NHS II* Nurse’s Health Study, *SDANA* Seven-day Adventists and non-Adventists, *YPAS* Yale Physical Activity Survey, *AAS* Active Australia Survey, *CAQ-PAI* College Alumnus Questionnaire Physical Activity Index, *GPPAQ* General practice physical activity questionnaire, *IPAQ-LF* International Physical Activity Questionnaire – Long Form, *IPAQ-SF* International Physical Activity Questionnaire – Short Form, *OSPAQ* Occupational Sitting & Physical Activity Questionnaire, *OSWEQ* Online Self-reported Walking and Exercise Questionnaire, *PASE* Physical Activity Scale for the Elderly, *SPAQ2* Scottish Physical Activity Questionnaire, *PAR* Stanford 7-day Physical Activity Recall, *TPAQ* Transport Physical Activity Questionnaire

**Table 9:** Overall rating of psychometric properties for each PAQ using the levels of evidence by Schellingerhout et al. (2011)

Assessment	Internal Consistency	Reliability Testing	Measurement Error	Content validity	Structural validity	Hypothesis testing
EPAQ2 *Usual 7-days*	NR	Conflicting	NR	Indeterminate	NR	Strong (Negative)
EPIC PAQ *Usual 7-days*	NR	Strong (Negative)	Indeterminate	Moderate (Negative)	NR	Conflicting
IPEQ-WA *Usual 7-days*	Indeterminate	Conflicting	NR	NR	Moderate (Positive)	Moderate (Positive)
NHS II *Usual 7-days*	NR	Moderate (Negative)	NR	NR	NR	Strong (Positive)
SDANA *Usual 7-days*	Indeterminate	Conflicting	NR	Moderate (Positive)	NR	Conflicting
Stanford Usual Activity Questionnaire *Usual 7-days*	NR	Moderate (Negative)	NR	NR	NR	Moderate (Negative)
YPAS *Usual 7 days*	NR	NR	NR	NR	NR	Strong (Negative)
AAS *Past 7-days*	NR	Strong (Negative)	NR	NR	NR	Moderate (Negative)
CAQ-PAI *Past 7-days*	Indeterminate	Strong (Negative)	NR	NR	NR	Strong (Negative)
Checklist Questionnaire *Past 7-days*	NR	NR	NR	Strong (Positive)	NR	Moderate (Negative)
GPPAQ *Past 7-days*	NR	Strong (Negative)	NR	NR	NR	Indeterminate
IPAQ-LF *Past 7-days*	NR	NR	NR	NR	NR	Indeterminate
IPAQ-SF *Past 7-days*	NR	Moderate (Negative)	NR	NR	NR	Strong (Negative)
IPAQ-SF (recall confidence) *Past 7-days*	NR	Strong (Negative)	NR	NR	NR	Strong (Negative)
IPEQ-W *Past 7-days*	Indeterminate	Conflicting	NR	NR	Moderate (Positive)	Moderate (Positive)
OSPAQ *Past 7-days*	NR	Strong (Positive)	NR	NR	NR	Conflicting
OSWEQ *Past 7-days*	NR	Conflicting	NR	Limited (Positive)	NR	Conflicting
PASE *Past 7-days*	Conflicting	Strong (Negative)	Indeterminate	Strong (Positive)	NR	Conflicting
PA Recall Instrument	NR	Moderate (Negative)	NR	NR	NR	Moderate (Negative)
SPAQ2 *Past 7-days*	NR	Limited (Positive)	NR	NR	NR	Moderate (Positive)
Stanford 7-day Physical Activity Recall (PAR) *Past 7-days*	Moderate (Positive)	Strong (Negative)	NR	NR	NR	Strong (Negative)
TPAQ *Past 7-days*	NR	Conflicting	NR	Moderate (Positive)	NR	Conflicting

*Notes*. Level of Evidence: Strong evidence positive/negative result (consistent findings in multiple studies of good methodological quality OR in one study of excellent methodological quality); Moderate evidence positive/negative results (consistent findings in multiple studies of fair methodological quality OR in one study of good methodological quality); Limited evidence positive/negative result (one study of fair methodological quality); Conflicting findings; Indeterminate = only indeterminate ratings on the measurement property (i.e., score = ? in Table 8); *NR* not reported

*EPAQ2* EPIC Physical Activity Questionnaire 2, *EPIC PAQ* EPIC Physical Activity Questionnaire, *IPEC-WA* Incidental and Planned Exercise Questionnaire for the Usual-week, *IPEC-W* Incidental and Planned Exercise Questionnaire for the Past-week, *NHS II* Nurse’s Health Study, *SDANA* Seven-day Adventists and non-Adventists, *YPAS* Yale Physical Activity Survey, *AAS* Active Australia Survey, *CAQ-PAI* College Alumnus Questionnaire Physical Activity Index, *GPPAQ* General practice physical activity questionnaire, *IPAQ-LF* International Physical Activity Questionnaire – Long Form, *IPAQ-SF* International Physical Activity Questionnaire – Short Form, *OSPAQ* Occupational Sitting & Physical Activity Questionnaire, *OSWEQ* Online Self-reported Walking and Exercise Questionnaire, *PASE* Physical Activity Scale for the Elderly, *SPAQ2* Scottish Physical Activity Questionnaire, *PAR* Stanford 7-day Physical Activity Recall, *TPAQ* Transport Physical Activity Questionnaire

## Discussion

The current review examined the methodological quality of a large number of studies examining 7-day PAQs and the psychometric quality of included PAQs. We identified 21 PAQs, of which seven were Past-week PAQs and 14 were Usual-week PAQs, which led to the retrieval of 44 corresponding original articles reporting on the psychometric properties of the included PAQs. According to the COSMIN taxonomy, reliability and *hypothesis testing* were the most commonly reported psychometric properties, while *internal consistency*, *measurement error*, *content validity* and *structural validity* were seldom examined. The methodological quality of the studies for PAQs was good to excellent although the overall quality of a majority of psychometric properties of PAQs showed “negative” ratings. According to the magnitude of the weighted mean r-values, Past-week PAQs appeared to have better convergent validity compared to Usual-week PAQs, although the overall psychometric qualities of both Past-week PAQs and Usual-week PAQs were weak. Despite weak overall psychometric qualities, IPEQ-WA had the greatest number of “moderate positive” ratings with no “negative” ratings for Usual-week PAQ. For the Past-week PAQs, IPEQ-W had the greatest number of “moderate positive” ratings with no “negative” ratings and SPAQ2 had “limited positive” to “moderate positive” ratings with no “negative” ratings. The overall finding, however, is that a substantial number of psychometric properties were either not reported or showed “moderate negative” to “strong negative” ratings irrespective of PAQ type.

### Quality of studies using the COSMIN taxonomy

According to the COSMIN taxonomy, the reliability domain consists of *internal consistency*, *reliability testing* and *measurement error* [15]. Of these psychometric properties, reliability testing was reported in a majority of PAQs, in the form of *test-retest reliability*, with the exception of three PAQs (YPAS, Checklist Questionnaire and IPAQ-LF). *Internal consistency* was only detailed in six PAQs (IPEQ-WA, SDANA, CAQ-PAI, IPEQ-W, PASE and PAR). Most of these PAQs showed moderate to excellent methodological quality for reliability testing, which are in line with previously published systematic reviews that have examined the methodological quality of self-reported PAQs in the adults [19] and elderly [21]. However, our current findings are in contrast to those reported by [16], where half of their ratings for the methodological quality of *test-retest reliability* were ‘fair’. These discrepancies could be due to the current review incorporating a modified COSMIN criteria by [26] which accounts for subtle differences in the psychometric quality of each study. Given that only few studies reported on *internal consistency* with 4 out of 7 COSMIN ratings scored as “indeterminate”, determining the quality of this psychometric property for Usual-week and Past-week PAQs is at present not possible in the current review.

Undoubtedly, the greatest deficiency for the reliability domain was the lack of examination of *measurement error*, which was only reported in two PAQs (EPIC PAQ and PASE) based on two studies[29, 30]. Not knowing the *measurement error* of a PAQ means that we cannot say with confidence that the reported PA level of a person is indeed accurate (i.e., a true reflection of the construct being measured). A framework to improve accuracy of PAQs has been published [10], although further studies are needed to determine the *measurement errors* of popular PAQs to provide practitioners and researchers with robust measures.

With respect to validity, *hypothesis testing* was reported in all PAQs with good to excellent study qualities. A majority of *hypothesis testing* involved studies assessing convergent validity of PAQs by comparing its properties with other comparator instruments (e.g., accelerometers). These results differ to those reported by previous reviews that examined the psychometric properties of PAQs in the adults and elderly [16, 19, 21] by reporting poor to fair study quality. Again, these discrepancies in findings may be attributed to differences in the types of criteria used to assess the psychometric qualities of PAQs. *Content validity* was seldom reported (only seven PAQs) although the study quality ranged from good to excellent. *Structural validity* was only assessed for two PAQs with good study qualities..In the current review, the quality for *structural validity* was not assessed in a majority of studies given that the underlying constructs of PAQs were not assessed using statistical analyses to determine the uni-dimensionality of subscales (e.g., factor analysis, principle component analysis, Rasch analysis). Only the IPEQ [13] incorporated factor analyses and Rasch analyses to determine the overall structure and measurement properties of IPEQ. Subsequently, caution should be taken as assessment of *internal consistency* and *structural validity* are only relevant when instruments form a reflective model (i.e., when items are indicative of the same underlying constructs), rather than a formative model (i.e., when items together form the construct). When exploring the underlying constructs of various PAQs, future research should address whether studies are based on a formative or reflective model.

### Quality of psychometric properties

A key aim of the current review was to examine the differences between Usual-week and Past-week PAQs. Previously, different recall versions of the IPEQ were examined in the one study [13] with IPEQ-WA (i.e., Usual-week PAQ) exhibiting greater *test-retest reliability* compared to the IPEQ-W (i.e., Past-week PAQ). This is not surprising, given that Usual-week PAQs control for week-to-week variation in PA patterns [10]. Interestingly, our findings showed comparable *test-retest reliability* between Usual-week PAQ and Past-week PAQ according to the magnitude of the weighted mean r-values. These discrepancies in findings between [13] (i.e., differences in *test-retest reliability* between IPEQ-W and IPEQ-WA) and the current review (i.e., similar *test-retest reliability* between Usual-week and Past-week PAQs) is possibly due to differences in acceptable cut-offs for *test-retest reliability*. For example, an ICC of ≥0.6 was considered as acceptable by [13], whereas ICC of ≤0.7 in the current review (based on use of the criteria by [17]) was below the acceptable cut-off and was therefore rated as “negative”.

Whilst comparable *test-retest reliability* was reported between Usual-week and Past-week PAQs in the current review, Past-week PAQs exhibited stronger *convergent validity* than Usual-week PAQs when compared against direct measures (e.g., accelerometers). Such findings are expected, since recall of Past-week PAQs typically coincide with data collected from direct measures during the past week. Subsequently, Past-week PAQs may be more accurate in reporting actual PA patterns than Usual-week PAQs. Whilst the magnitude of weighted r-values between PAQs with direct measures and PAQs with indirect measures were similar for Usual-week PAQs (both were in the weak range), there was a moderate relationship between Past-week PAQs and direct measures whilst a weak relationship shown between Past-week PAQs and indirect measures. Accordingly, while it would be expected that individuals who reported higher levels of physical activity would demonstrate greater physical fitness, determining the validity of PAQs with indirect measures may not be as appropriate as direct measures, given that the dimension of measures are different [31] (e.g., two different types of measures that report level of PA would be more similar than measures that report level of PA and physical fitness).

For the overall psychometric qualities, only minor differences were evident between the PAQs. However, for each recall method, the strongest PAQ identified according to psychometric quality was IPEQ-WA for Usual-week PAQs and IPEQ-W for Past-week PAQs given that 4 out of 6 psychometric properties were evaluated of which *structural validity* and *hypothesis testing* had “moderate positive” results. However, *internal consistency* and *reliability* had “indeterminate” and “conflicting” results, respectively, warranting further research in the psychometric properties of IPEQ-WA and IPEQ-W. Furthermore, SPAQ2 indicated positive ratings for *reliability testing* and *hypothesis testing*, demonstrating good validity and reliability of Past-week PAQ. However, only two psychometric properties were assessed for SPAQ2 which appears to be a common limitation for all included PAQs. Subsequently, future studies should assess other psychometric properties to determine the overall quality of PAQs.

While a majority of PAQs consisted of *reliability testing* and *hypothesis testing*, irrespective of recall methods, these psychometric properties also had the most number of “moderate negative” to “strong negative” ratings. These findings are in line with findings from other systematic reviews that have reported the psychometric qualities of self-reported PAQs, even though these reviews were smaller in scope [16, 19, 21]. Interestingly, the findings from the current systematic review, and of others [16, 19, 21], conflict with interpretations of the quality of reported validity and reliability values of PAQs as reported and interpreted by the authors themselves in a majority of included studies. This is because many of the authors in the included studies have interpreted *test-retest reliability* and *convergent validity* as being acceptable based on associations reported at a statistically significant level, with minimal regard to the *strength* of the relationship. According to previously established and accepted criteria [17, 18, 26], acceptable *test-retest reliability* for correlations (r or rho) and ICC were 0.8 and 0.7, respectively. Furthermore, convergent validity of a questionnaire is acceptable if the correlation with its comparator instrument is at a statistically significant level (*p* ≤ 0.05) and the strength of the correlation is at least moderate (*r* ≥ 0.5) [17, 18, 26]. Accordingly, whilst the included studies reported associations at a statistically significant level for both *reliability testing* and *hypothesis testing*, the results were classified as “negative” ratings in the current review given that the magnitude of the association was not met in accordance to the psychometric criteria (i.e., *r* ≥ 0.5). Consideration for the strength of the relationship is essential, given that a large sample size will exhibit associations at a statistically significant level, despite weak associations, as reported in a number of studies included in the current review. Indeed, an appropriate sample size must be met for studies exploring psychometric properties of instruments in order to reach clinically relevant conclusions, given that a limited sample size may not be generalisable to a wider population [32]. Furthermore, future studies should interpret correlations based on the magnitude of the correlation, rather than the statistical significance (i.e., *p* ≤ 0.05) when determining validity of PAQs [32]. Subsequently, interpretation regarding validity and reliability of PAQs should consider both the statistical significance and the corresponding magnitude of the association between measured variables.

### Limitation

There are a number of limitations that should be elaborated upon. First, the PAQs with recall timeframes other than 7-days were outside the scope of this systematic review and may have different psychometric properties. Second, the PAQs in the current review were limited to those used by English speaking adults and those that were self-reported. Future studies may compare different recall methods of PAQs using other populations (e.g., children, individuals from non-English speaking backgrounds, etc.) and different PA collection methods (e.g., PAQs with recall time frames other than 7-day periods, studies that administered PAQs as interviews etc.). Fourth, the PAQs selected for the current review is one of energy expenditure. It is important to acknowledge that PA level can be influenced by social, physical and policy environments [33, 34]. Subsequently, further research is warranted to analyse the psychometric properties of other PAQs that account for these factors. Finally, while evaluation of *responsiveness* was beyond the scope of the current review, comparison of this psychometric property between different PAQ types may support the suitability of PAQs to assess PA level.

## Conclusion

In conclusion, the current review identified that most PAQs did not report on several psychometric properties. Based upon well-defined analyses, the overall psychometric quality of PAQs showed multiple “negative” ratings, indicating that current 7-day PAQs are rather weak and caution should be taken when interpreting PA level using these PAQs. When comparing different recall methods, Past-week PAQs showed a stronger correlation with direct measures compared to that of Usual-week PAQs, suggesting that Past-week PAQs may be a more accurate measure of PA patterns. However, minimal differences were noted between the Usual-week and Past-week PAQs for the overall psychometric quality. While IPEQ-W and IPEQ-WA demonstrated the strongest psychometric properties with positive ratings, followed by SPAQ2, there were still a substantial number of psychometric qualities that were not assessed which limits the usability of these PAQs. To resolve the issues identified in the current review, future studies are encouraged to investigate a greater range of psychometric properties for those 7-day PAQs that are promising (e.g., IPEQ-WA, IPEQ-W and SPAQ2). However, further investigation is warranted for all 7-day PAQs with ‘negative’ ratings by incorporating item response theory.

## Abbreviations

AAS: Active Australia survey
CAQ-PAI: College alumnus questionnaire physical activity index
COSMIN: Consensus-based standards for the selection of health measurement instrument
EPAQ2: EPIC physical activity questionnaire 2
EPIC PAQ: EPIC physical activity questionnaire
GPPAQ: General practice physical activity questionnaire
ICC: Intra-class correlation coefficient
IPAQ-LF: International physical activity questionnaire – long form
IPAQ-SF: International physical activity questionnaire – short form
IPEC-W: Incidental and planned exercise questionnaire for the past-week
IPEC-WA: Incidental and planned exercise questionnaire for the usual-week
IPEQ: Incidental and planned exercise questionnaire
NE: Not evaluated
NHS II: Nurse’s health study
OSPAQ: Occupational sitting & physical activity questionnaire
OSWEQ: Online self-reported walking and exercise questionnaire
PA: Physical activity
PAQ: Physical activity questionnaire
PAR: Stanford 7-day physical activity recall
PASE: Physical activity scale for the elderly
SDANA: Seven-day adventists and non-adventists
SPAQ2: Scottish physical activity questionnaire
TPAQ: Transport physical activity questionnaire
W: Past-week physical activity
WA: Average weekly physical activity
YPAS: Yale physical activity survey
